# Obesity, Physical Fitness and Inflammation in the Elderly

**DOI:** 10.3390/geriatrics2040030

**Published:** 2017-09-21

**Authors:** Bruno Silva, Miguel Camões, Mário Simões, Pedro Bezerra

**Affiliations:** 1 School of Sport and Leisure—Viana do Castelo Polytechnic Institute, 4900-347 Viana do Castelo, Portugal; joaocamoes@esdl.ipvc.pt; 2 Maia Polytechnic Institute, Portugal—Grupo de Investigação para o Desporto, Educação e Saúde (GIDES), 4475-690 Maia, Portugal; msimoes@esdl.ipvc.pt; 3 Research Center in Sports Sciences, Health and Human Development (CIDESD), 5000-801 Vila Real, Portugal; pbezerra@esdl.ipvc.pt

**Keywords:** adiposity, muscle strength, vitamin D, C-reactive protein

## Abstract

Among the elderly, obesity is paradoxically associated with a lower mortality risk. Thus, this study describes fitness levels by Body Mass Index (BMI) category and the associations of high-sensitivity C-reactive protein (hs-CRP) and Vitamin D levels with muscle strength, in community-dwelling older adults. A cross-sectional study, with 1338 subjects having mean age of 78.3 years, were assessed in anthropometrics, muscle strength, and cardiorespiratory fitness. In a sub-sample, blood samples were collected and objective markers of inflammation were provided: high-sensitivity C-reactive protein (hs-CRP) and Vitamin D (25(OH) D). Obese women (BMI ≥ 30.0 kg/m^2^) showed significantly better results for grip strength than normal weight group (BMI between 18.5–24.9 kg/m^2^): 22.3 (7.0) vs. 20.0 (6.8); *p* = 0.002. After adjustment, higher levels of hs-CRP were an independent predictor of lower levels of grip strength (β = −0.213, 95% CI: −0.424; −0.002) and Vitamin D levels were positively associated with higher levels of muscle strength (β = 0.098, 95% CI: 0.008–0.189). The multivariate analysis found a significant and positive association between 25(OH) D and grip strength: (β = 0.098, 95% CI: 0.008–0.189). A positive pattern of higher levels of absolute strength among obese older subjects could have an important impact on morbidity and mortality risk, through the inverse association with acute inflammation and an increase in Vitamin D profile.

## 1. Introduction

The changing of body composition is an important hallmark of the ageing process, namely the consequences of the central obesity incidence [1,2]. This fact has transverse effects in functional capacity, muscle mass, and strength, disabilities in activities of daily living and impaired quality of life in elderly population [3,4,5,6]. In Portugal, 19% of the population has 65 or more years [7], with recognized implications for society organization, economy, and public health policies. This prevalence is not homogeneous throughout the country, and some regions present alarming proportions of older subjects. In the case of Viana do Castelo, fifty percent of the elderly population they have are more than 75 years old [7]. With ageing being an important determinant of central and overall obesity development [1], these facts conduct to an alarming prevalence and incidence estimates among Portuguese habitants [8,9]. Nevertheless, and despite the adverse effects that obesity has on the risk factors associated with cardiovascular diseases, morbidity, and quality of life [10,11,12], association of excess weight with increased mortality is substantially weaker in elderly when compared with data among younger people [5,10,13]. The causes of increased mortality are essentially the same as those found among younger adults. However in the elderly, higher BMI levels appear to have no association with higher mortality or even to provide some survival advantage, when compared to low Body Mass Index (BMI) levels [14]. Using BMI as a risk factor to health, the optimal BMI range for the lowest mortality risk is between 25–30 kg/m^2^ (overweight) [5,10,11,15,16,17]. This aspect has been termed the ‘obesity paradox’. With ageing, typically offset by gains in fat mass are normally associated with the declines on lean muscle mass in about 25% between 75 to 80 years old [18]. Consequently, these conducts to important age related changes, with decreasing functional activities of the daily living and independency [19]. In the elderly, muscle strength is an important determinant of functional limitation and poor health [5]. As already described, muscle strength, estimated via the handgrip test, is an independent determinant of mortality [20]. Likewise, low levels of physical fitness represent poor results in handgrip strength [21]. In another spectrum, the physical activity practice, and performance are also associated with inflammatory biomarkers [22]. The data from these authors suggest that reduced inflammation is associated with an increased physical fitness performance and may be all so associated with lesser degree of central obesity. Consistent associations were found between physical activity and performance, lower erythrocyte sedimentation rate, and lower plasma levels of fibrinogen, C-reactive protein, and interleukin [23]. However, obesity is a consequence of many risk factors and the origin of inflammation during obesity and the underlying molecular mechanisms that explain its occurrence are not fully understood [24]. Despite the well-established life course epidemiological trends on obesity and physical fitness among adults, data are still needed to establish the potential mechanisms that could partially explain the obesity paradox among older subjects, through the objective multivariate analysis of the fitness impact on the inflammatory profile of these individuals. Therefore, this study aimed to describe the relationship between overweight/obesity and physical fitness, and test the independent associations of high-sensitivity C-reactive protein (hs-CRP) and VitaminD (25(OH) D) levels with strength, in non-institutionalized older adults.

## 2. Materials and Methods 

### 2.1. Participants

A cross-sectional study among the elderly population, more than 70 years old, recruited from Viana do Castelo region, north of Portugal. A total of 1338 subjects (69.2% women), with a mean (sd) age of 78.3 (6.2) years, were evaluated between October of 2013 and April of 2014 (Table 1). The subjects were volunteers, from daily living center, public and private nursing homes, and participants in exercise sessions and social events. Subjects were asked about their physical activity routines and allocated to Exercise and no Exercise groups. This measurement was assessed by the answer to the question: “Do you practice sports or physical activity sufficient to produce sweating or shortness of breath?” [25]. An exercise group was defined as individuals who respond “Yes” to question and assume to do it at least two times a week, systematically. No exercise group includes individuals reporting either no exercising or exercising less than two times a week, systematically.

In a randomly recruited sub-sample of 91 non-institutionalized older adults (77% female; aged 70–94 years), blood samples with objective markers of inflammation were collected.

### 2.2. Data Collection

The study was approved by the Human Research Ethics Committee of the Polytechnic Institute of Viana do Castelo (PTDC/DTP-DES/0209/2012) and was carried out in full compliance with the Declaration of Helsinki [23]. Written informed consent was obtained from each participant. Before anthropometric and physical fitness testing, participants completed a structured questionnaire with socio-demographic and behaviour characteristics, carried out by trained professionals.

### 2.3. Anthropometrics

Anthropometrics were obtained with the participant wearing light clothing and no footwear. Body weight was measured on a scale (SECA 760, seca gmbh & co., Hamburg, Germany) to the nearest 500 g, and height to the nearest 0.1 cm in a standing position using a portable stadiometer (SECA 217, seca gmbh & co., Hamburg, Germany).

BMI was calculated (kg/m^2^), and the individuals were categorized according to the WHO International classification [26]: Normal Weight—BMI between 18.5 and 24.9 kg/m^2^; Overweight—BMI between 25.0–29.9 kg/m^2^; and Obesity—BMI ≥ 30.0 kg/m^2^.

### 2.4. Physical Fitness

Muscle strength was assessed by a Handgrip Test (HandT). The handgrip strength (kilograms) was measured using a hydraulic hand dynamometer (SH5001, SAEHAN Corporation, Changwon, South Korea), according to the American Society of Hand Therapists [27]. Three measures were collected in both hands with an interval of 30 s between each and considered for analyses the maximum obtained value. Relative muscle strength was calculated with the following formula: relative strength = absolute strength (HandT)/body mass (kg).

The Cardiorespiratory Fitness was evaluated by the 6-min walking test (6 MW), and performed according to the American Thoracic Society [28]. The cardiorespiratory scores were expressed in meters (m) and participants were instructed to walk according to a path, as quickly as possible, continuously for 6 min. 

### 2.5. Inflammation

As markers of inflammation, high-sensitivity C-reactive protein (hs-CRP) and Vitamin D (25(OH) D) were collected in the serum of the blood samples with 12 h overnight fasting. Vitamin D (25(OH) D) was evaluated by immunonephelometric and C-reactive protein levels were determined by a particle enhanced turbid metric method. 

### 2.6. Statistical Analysis

Due to the type of the variable of distribution, it was necessary to use non-parametric tests. The Kruskal-Wallis test was applied to assess the differences between the levels of physical fitness among the weight classes. Subsequently, it was necessary the post hoc Dunnett’s T3 test, to establish where the differences occurred, among the groups analysed and the Chi Square test to assess thr differences between proportions. The significance level was set at *p* < 0.05.

Statistically significant differences in gender distribution between weight classes were observed (16.8%, 53.8%, and 29.0% of males among normal weight, overweight, and obesity categories, respectively), and established stratification by gender, to describe the values of physical fitness between weight categories. A generalized linear model was created to study the independent associations of acute inflammation (hs-CRP) and Vitamin D, with maximal handgrip muscle strength (Kg), computing beta coefficients, and 95% confidence intervals (95% CI). All statistical analysis was performed with IBM SPSS version 19 for Windows.

## 3. Results

Regarding the physical fitness parameters according to the BMI categories (Table 2), in women, the NW group presented lower results in muscle strength when compared to the other groups, in which the obesity (OB) group had higher median values for handgrip strength (22.3 vs. 20.0, *p* = 0.002). In respect to the cardiorespiratory fitness, a reversed relationship was observed, since the normal weight (NW) group presented significant better results than other groups. After adjusting strength for body weight (strength to weight ratio), the NW group showed a statistically significant better pattern of relative strength, when compared to the other groups.

Among the men, those in the OW group had higher scores in muscle strength, than the other groups, while for cardiorespiratory capacity, like female, higher median values were observed among the NW group (*p* = 0.042). However, the results were statistically significant only among the group of NW and OB for the 6MWT (NW = 418.5 (181.0); OW = 406.0 (176.5); OB = 376.0 (138.3)) (Table 3). When adjusting strength for body weight, the relative strength results across categories of BMI, are similar to the previously described among women. 

Within the subsample of 91 elderly participants (Table 4), Maximal Handgrip Strength is positively associated with gender (*B* = 8.560, 95% CI: 5.894; 11.225), 6MW (*B* = 0.028, 95% CI: 0.018; 0.038) and Vitamin D (*B* = 0.098, 95% CI: 0.008; 0.189), all significant, and negatively associated to hs-CRP (*B* = −0.213, 95% CI: −0.424; −0.002), independent of age and BMI.

## 4. Discussion

Overweight and obese elderly women showed better results of absolute strength than the normal weight group. Despite these better performances on strength, elderly subjects with a BMI above 25 kg/m^2^ showed consistently worse results in the cardiorespiratory fitness test. In addition, after adjustment of several confounders, maximal strength was inversely associated with acute inflammation and with an increase in Vitamin D serum profile.

The lower scores of cardiorespiratory fitness and higher levels of BMI among these individuals probably conducts them to an increased risk of morbidity and mortality, such as observed in other studies [29,30]. Although, together with age, body composition, and strength influence the 6-min walking distance in obese subjects [17]. Observing muscle strength scores, the dependency association is different than the one with cardiorespiratory fitness. Women in the OB group always have better scores than the ones in the other groups. Similar results were achieved in a longitudinal study [31], where the obese (high fatness) had better results in muscle strength than the other groups. In men, the association of muscle strength scores and BMI groups is different, with the OW group presenting the better results that are not statistically significant. These differences may be related to the differences in body composition between sexes and the effects of BMI on performance among women when compared with men, because after the adjustment for age and height, a higher BMI is normally associated with stronger grip strength only among men [32]. Another fact is the need for muscle strength for mobility-related activities increased along with BMI, particularly in men [33].

In our sample, and when comparing men and women, these associations were not confirmed, because women in the OB group and men in the overweight (OW) group had the better scores in muscle strength. This may be associated with the different declines in muscle strength between men and women, and a more rapid loss of muscle strength than the concomitant loss of muscle mass [34]. Also, weight is positively associated with muscle strength [35] and women tend to have a lower proportion of lean mass than males [32]. Despite this association between BMI and strength found in literature, our study provides important observational data among elderly subjects, reporting a significant association between strength and biomarkers of inflammation, conferring some protection patterns, independent of BMI. Similar findings were reported in one cross-sectional and longitudinal studies demonstrate associations between physical activity and the decrease in inflammatory markers, especially in the elderly, suggesting a positive influence of physical activity in reducing the levels of inflammatory markers [36,37]. In our specific populations, this association is also related to obesity levels, as measured with BMI.

Those in the OW group, in both sexes, consistently show better results regarding muscle strength than the group NO. However, given that lower strength indexes are related to an increased susceptibility of falls [38,39], in this population, and observing only this factor, it appears that the group of OW elderly, have less of a downside risk than those with a BMI less than 25 kg/m^2^. Furthermore, having 70 years and more at baseline measure, the risk of death increased with decreasing handgrip strength for each BMI category [40]. 

Cardiorespiratory fitness does not seem to be a major factor when analyzing the determinants that mediate the obesity and mortality in the elderly, among the studied population. On the other hand, muscle strength, especially among elderly men, seems to be an important indicator in understanding the best survival rates of those with BMI > 25 kg/m^2^, giving consistency to some of the observational studies [41]. 

Still, observing the strength to weight ratio (relative strength), the data indicate that the NW group and the OW group presents significantly higher strength and cardiorespiratory fitness than the orders of the OB group, and the NW group better than the OW group. This may suggest that taking into consideration the changes in body composition in the physiological ageing process may be important to consider the strength to weight ratio (relative strength) when assessing muscle strength.

The cross-sectional design and sample size, particularly when the sample is fractionated by gender, limit the interpretation of the results, losing power in comparisons between groups, namely among men. However, the stratification by sex, the homogeneity of ages, the prevalence of exercise training between BMI categories, and the most in a community context, support the observation. In addition, sexes were pooled in the regression analysis to increase the strength of the estimations. Objective measures of the exposures and the main outcomes of interest were accomplished by trained physicians supported in reference methods, increasing the quality of the observational data. Furthermore, we know that the use of BMI does not match the Gold Standard anthropometric measurements, because it does not allow the discrimination of body composition. However, it is an easy and common measure that may have different physiological meanings and distinct associations with adverse health consequences [42]. Other potential confounders have not been evaluated, limiting inferences inherent to the exposed dependent relationship.

## 5. Conclusions

A positive pattern of higher levels of absolute strength among obese older subjects could have an important impact on morbidity and mortality risk, through the inverse association with acute inflammation and an increase on Vitamin D profile.

## Figures and Tables

**Table 1:** Participant’s socio-demographic and behavioural characteristics, according to Body Mass Index (BMI) categories.

	BMI			*p* Value
	Normal Weight	Overweight	Obese	
**n (%)**	234 (17.5)	590 (44.1)	514 (38.4)	
**Men**	69 (16.8)	221 (53.8)	122 (29.0)	<0.001
**Women**	165 (17.7)	369 (39.8)	392 (42.4)	
**Institution**	47 (20.1)	116 (19.7)	107 (20.8)	0.892
**Community**	187 (79.9)	474 (80.3)	407 (79.2)	
**Exercise**	139 (59.4)	364 (61.7)	308 (59.9)	0.761
**No exercise**	95 (40.6)	225 (38.1)	206 (40.1)	
**Single**	26 (11.1)	53 (9.0)	43 (8.4)	0.211
**Married**	105 (44.9)	241 (40.8)	191 (37.2)	
**Widowed**	98 (41.9)	278 (47.1)	268 (52.1)	
**Divorced**	5 (2.1)	18 (3.1)	12 (2.3)	
**Mean (sd)**				
**Age (years)**	79.4 (6.7)	78.1 (5.9)	77.6 (5.5)	<0.001
**Weight (kg)**	56.4 (7.5)	68.1 (8.0)	81.0 (12.4)	<0.001
**Waist Circumference (cm)**	84.3 (8.0)	97.9 (42.7)	108.8 (50.4)	<0.001
**Hip Circumference (cm)**	95.0 (6.4)	101.8 (7.3)	112.9 (9.8)	<0.001
**Arm Circumference (cm)**	26.3 (2.3)	29.0 (2.4)	32.3 (3.0)	<0.001
**Thigh Circumference (cm)**	46.1 (7.6)	68.1 (8.0)	54.1 (9.4)	<0.001

Normal Weight—BMI between 18.5 and 24.9 kg/m^2^; Overweight—BMI between 25.0–29.9 kg/m^2^; Obesity—BMI ≥ 30.0 kg/m^2^.

**Table 2:** Relationship between physical fitness and weight classes, in women.

Median (Range)	Normal Weight	Overweight	Obesity
**Handgrip Test (kilograms)**	20.00 (6.8)	21.8 (8.0)	22.3 (7.0) **
**Strength to weight ratio**	0.38 (0.12) ^†^	0.35 (0.12) ^††^	0.29 (0.09)
**6 Min Walk Test (meters)**	353.2 (215.6) *	346.0 (190.0)	311.0 (212.3)
**Distance to weight ratio**	6.6 (3.92) ^†^	5.3 (2.99) ^††^	4.1 (2.77)

Normal Weight—BMI between 18.5 and 24.9 kg/m^2^; Overweight—BMI between 25.0–29.9 kg/m^2^; Obesity—BMI ≥ 30.0 kg/m^2^. * Statistical significant differences between Normal Weight group and Obesity group; ** Statistical significant differences between Obesity group and Normal Weight group; ^†^ Statistical significant differences between Normal Weight group and Overweight and Obese groups; ^††^ Statistical significant differences between Overweight group and Obesity group.

**Table 3:** Relationship between physical fitness and weight classes, in men.

Median (Range)	Normal Weight (NW)	Overweight (OW)	Obesity (OB)
**Handgrip Test (kg)**	31.5 (12.8)	33.0 (12.0)	32.0 (13.0)
**Strength to weight ratio**	0.51 (0.17) **	0.45 (0.14) ***	0.36 (0.15)
**6 Min Walk Test (meters)**	418.5 (181.0) *	406.0 (176.5)	376.0 (138.3)
**Distance to weight ratio**	6.9 (2.70) **	5.5 (2.23) ***	4.2 (1.82)

Normal Weight—BMI between 18.5 and 24.9 kg/m^2^; Overweight—BMI between 25.0–29.9 kg/m^2^; and Obesity—BMI ≥ 30.0 kg/m^2^. * Statistical significant differences between Normal Weight group and Obesity group; ** Statistical significant differences between Normal Weight group and Overweight and Obesity groups; *** Statistical significant differences between Overweight group and Obesity group.

**Table 4:** Modelling the associations between muscle strength and cardiovascular determinants.

Parameter	Beta (*B*)	*p*-Value	95% CI
Gender	8.560	<0.001	5.894; 11.225
Age (years)	−0.022	0.827	−0.219; 0.175
BMI (kg/m^2^)	0.182	0.220	−0.111; 0.474
6 min Walking Test (meters)	0.028	<0.001	0.018; 0.038
C-reactive protein—Hs-CRP (mg/L)	−0.213	0.048	−0.424; −0.002
Vitamin D—25(OH) D (ng/mL)	0.098	0.034	0.008; 0.189

Dependent Variable: Maximal Handgrip Strength.
